# Increased Risk of Type 1 Diabetes in Boys Under the Age of 5 Years During COVID‐19 Lockdowns in Finland, Sweden and Stanford, CA, USA—An Observational Multicenter Study

**DOI:** 10.1002/dmrr.70084

**Published:** 2025-09-01

**Authors:** Susanna Tall, Priya Prahalad, Martin Adiels, Annika Rosengren, Suvi M. Virtanen, David M. Maahs, Mikael Knip

**Affiliations:** ^1^ Research Programs Unit Faculty of Medicine University of Helsinki Helsinki Finland; ^2^ Population Department Finnish Institute for Health and Welfare Helsinki Finland; ^3^ Division of Pediatric Endocrinology Stanford University Stanford California USA; ^4^ Stanford Diabetes Research Center Stanford California USA; ^5^ Department of Molecular and Clinical Medicine Sahlgrenska Academy University of Gothenburg Gothenburg Sweden; ^6^ School of Public Health and Community Medicine Sahlgrenska Academy University of Gothenburg Gothenburg Sweden; ^7^ Sahlgrenska University Hospital/Ostra Västra Götaland Region Gothenburg Sweden; ^8^ Faculty of Social Sciences/Health Sciences Unit Tampere University Tampere Finland; ^9^ The Science Center of Pirkanmaa Hospital District Tampere Finland; ^10^ Department of Pediatrics Tampere University Hospital Tampere Finland; ^11^ Pediatric Research Center New Children's Hospital Helsinki University Hospital Helsinki Finland

**Keywords:** COVID‐19 pandemic, incidence, lockdown, SARS‐CoV‐2, type 1 diabetes

## Abstract

**Aims:**

The COVID‐19 pandemic has been associated with an increased incidence of type 1 diabetes. Changes in the type 1 diabetes incidences in countries like Sweden where very mild COVID‐19 pandemic related measures were applied have not been established so far. We analysed the incidence of type 1 diabetes during the COVID‐19 lockdown and before the lockdown in Sweden, Finland and Stanford, CA, USA.

**Materials and Methods:**

Type 1 diabetes incidence rates during the first 18 months of the SARS‐CoV‐2 pandemic (3/2020–8/2021) were compared to a period before the pandemic (three corresponding 18‐month terms 2014–2019) in Sweden, Finland and Stanford.

**Results:**

In Sweden, type 1 diabetes incidence increased by 5% (IRR 1.05; 95% CI = 0.98–1.12; *p* = 0.18, *N* = 4458), in Finland by 17% (IRR 1.17; 95% CI = 1.07–1.27; *p* < 0.001, *N* = 2881) and in Stanford by 10% (IRR = 1.10; 95% CI = 0.91–1.34; *p* = 0.34, *N* = 531) during the lockdown compared to the time before lockdown. In boys under 5 years of age, the incidence increased significantly in all regions: Sweden (IRR 1.21; 95% CI = 1.00–1.46; *p* = 0.05, *N* = 521), Finland (IRR = 1.33; 95% Cl = 1.06–1.67, *p* = 0.02, *N* = 363) and Stanford CA, USA (IRR = 2.07"; 95% Cl = 1.06–4.02, *p* = 0.03, *N* = 37).

**Conclusions:**

Lockdowns during the COVID‐19 pandemic may have untoward consequences such as an increased risk of type 1 diabetes in young children, boys in particular. The hygiene hypothesis may explain this finding.

## Graphical abstract

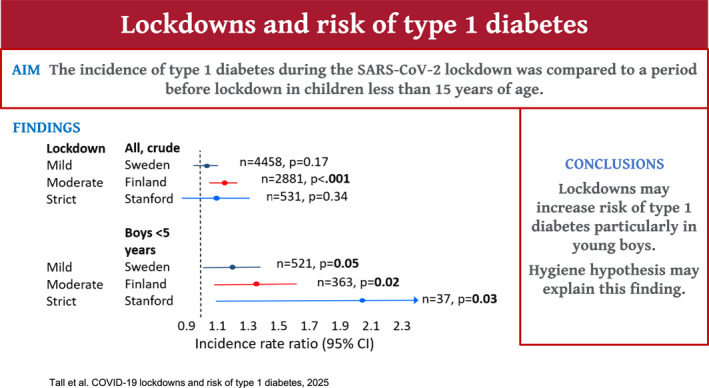

## 1 Introduction

During the COVID‐19 pandemic, transmission of SARS‐CoV‐2 was a global concern, resulting in extensive lockdowns [1]. Sweden was among the few countries that did not enforce strict lockdown measures but instead relied on voluntary mitigation recommendations [1, 2]. e.g., face masks were never mandatory except in clinical and care settings, daycare centres were open as usual and participation in classroom teaching in primary and secondary schools was compulsory throughout the pandemic [2]. In contrast, in Finland, the neighbouring country, many national and regional legal restrictions including partial daycare and school closures, and a strong recommendation of face mask usage were in place [3]. Many countries and regions, including many parts of the USA, have applied strict lockdown actions with full daycare and school closures, compulsory social distancing and mandatory face mask usage [4].

A radical decrease in SARS‐CoV‐2 and other contagious infections was observed during COVID‐19 related lockdowns in a series of different countries [4]. However, because of mild lockdown measures, Sweden had approximately 5 times more COVID‐19 infections compared to Finland, and other Nordic countries by the end of September 2021 [5]. While rates of contagious infections decreased during the lockdowns, a systematic and significant increase in the incidence of type 1 diabetes was observed in various countries [6].

The reason behind the observed increase in the type 1 diabetes incidence rates during lockdowns remains open [6]. Some research suggests that the SARS‐CoV‐2 virus might directly induce type 1 diabetes. A direct effect has been proposed, either through damage to the pancreatic *β* cells or through acceleration or precipitation of the disease process leading to type 1 diabetes by the SARS‐CoV‐2 virus. However, some studies have shown that the prevalence of SARS‐CoV‐2 antibodies is not different between those who developed type 1 diabetes during the acute pandemic and those who did not (e.g. [7]). These findings suggest that SARSCoV‐2 does not directly induce type 1 diabetes.

The observed increase in type 1 diabetes rates during lockdowns could also result from social distancing. The hygiene hypothesis states that the early exposure to infections may protect from type 1 diabetes [8]. In accordance, the decreased contacts with both pathogens and other microbes during the lockdowns have been proposed to explain the increase in type 1 diabetes rates [9]. However, several studies have reported that early viral infections may instead trigger type 1 diabetes (e.g. [10]). Therefore, the biodiversity hypothesis has largely replaced the hygiene hypothesis in the scientific literature. Briefly, the biodiversity hypothesis proposes that diversity of microbial species encountered via contact with nature enrich the microbiota in humans, which is the key to a balanced development of the immune system [11]. Nevertheless, COVID‐19 lockdowns and social isolation radically reduced infection rates, thereby providing a unique opportunity to further examine whether the hygiene hypothesis holds true [12]. So far, changes in the type 1 diabetes incidences in countries like Sweden where very mild COVID‐19 pandemic related lockdown measures were applied have not been compared with those of other countries applying stricter measures with subsequent reduced infections.

We established the incidences of type 1 diabetes during and before the pandemic in Sweden, where only voluntary mitigation recommendations were used and compared the findings to the changes in the incidences in Finland, and Stanford, CA, USA, where stricter lockdown actions were applied. Based on the hygiene hypothesis and previous research, we expected that out of the three included locations, the rates of type 1 diabetes would have increased least in Sweden.

## 2 Methods

The incidence of type 1 diabetes during the first 18 months of the SARS‐CoV‐2 pandemic was compared to the period before the pandemic in children under 15 years of age in Sweden, Finland and Stanford CA, USA. The study period for the pandemic comprises 18 months from March 1, 2020 to August 31, 2021. The reference period comprises three corresponding 18‐month terms 2014–2019 (from March 1, 2014, to Aug 31, 2015; March 1, 2016, to Aug 31, 2017; and March 1, 2018, to Aug 31, 2019).

The COVID‐19 pandemic period started on March 1, 2020 when severe social distancing regulations took place in Finland and Stanford and less so in Sweden [2, 3, 4]. The distancing regulations were adjusted depending on the prevailing availability in medical intensive care units, COVID‐19 incidence statistics, and the preferences of the local authorities [2, 3, 4]. Some social distancing regulations were in place throughout the pandemic study period in all the included study locations. August 31, 2021 was set as the ending point of the pandemic period as a new school year with classroom teaching started leading to more social contacts and increased infection rates among children in Autumn 2021. In addition, lockdown measures were relieved in August–September 2021 in Finland and Stanford.

### 2.1 Sweden

The National Diabetes Registry (NDR) in Sweden, initiated in 1996, is a nationwide tool for quality control of diabetes care in Sweden, with physicians and nurses annually reporting data from hospital outpatient clinics and primary care centres. The NDR contains information on almost all patients diagnosed with diabetes in Sweden. The national registry Swediabkids, was initiated in 2000, with information about diabetes in affected children and adolescents, including type of diabetes, and with almost full coverage from 2007. Since 2018, both registries are combined into the NDR. New incident cases of type 1 diabetes were captured from the NDR using the predefined categories based on ICD codes (E10).

### 2.2 Finland

We used the Finnish Paediatric Diabetes Register (FPDR), which contains data from all paediatric units taking care of children with newly diagnosed diabetes in Finland. The register covers more than 90% of such children [13]. Each unit contributes to the register with data collected using a structured questionnaire and with biological samples from all newly diagnosed children with parental consent to participate [14]. Children aged 10 years or older are also asked for informed consent. Approximately 99% of the participants were identified as having type 1 diabetes, diagnosed based on clinical characteristics and autoantibody status, and more than 98% were White. Children with confirmed monogenic diabetes were excluded as well as those with type 2 diabetes and other forms of diabetes.

### 2.3 Stanford

Counts of children developing type 1 diabetes during the periods of interest were obtained from an internal clinical diabetes registry. The diabetes registry included youth who had a clinical visit at Stanford Medicine Children's Health. The diabetes diagnosis was based on clinical symptoms and such characteristics as blood glucose testing, A1c, urine glucose, ketones, family history and islet autoantibody status. The overall paediatric population of the immediate catchment area for Stanford Medicine Children's Health was obtained from the State of California Department of Finance website [15]. The population data for California were downloaded and queried for the estimated paediatric populations of San Mateo County and Santa Clara County, the two counties closest to Stanford.

The diagnostic criteria for type 1 diabetes were based on WHO guidelines in all study locations [16].

### 2.4 Statistical Analysis

Statistical analyses were carried out using SAS 9.4, SAS Enterprise Guide for Windows version 7.1 A two‐tailed *p* value of 0.05 or less was considered significant.

The incidence of type 1 diabetes during the lockdown was statistically compared to the time before the lockdown in Sweden, Finland, and Stanford, CA. We calculated overall, sex‐specific, and age‐specific incidence rates of type 1 diabetes per 100,000 person‐years during the lockdown and before the lockdown period. We separated the participants into three age categories: < 5 years, 5 to < 10 years, and 10 to < 15 years. To quantify the possible change during the lockdown period compared to the reference period (before lockdown), we assessed incidence rate ratios (IRR) and their 95% confidence intervals (95% CIs). The incidence rate ratios and their 95% CIs were assessed by fitting multiplicative Poisson regression models to the number of children diagnosed with type 1 diabetes, when using the natural logarithm of person‐years as an offset term. Age at diagnosis and sex were assumed to be confounding factors and the adjusted *p* value for the IRR combined for all age groups and both sexes was calculated. As a sensitivity analysis, we compared the incidence during the lockdown to the most recent preceding 18‐month period (March 1, 2018, to Aug 31, 2019) and assessed the IRR when compared to the lockdown period.

## 3 Results

### 3.1 Incidence Rates and Incidence Rate Ratios—All Age Groups

**FIGURE 1:**
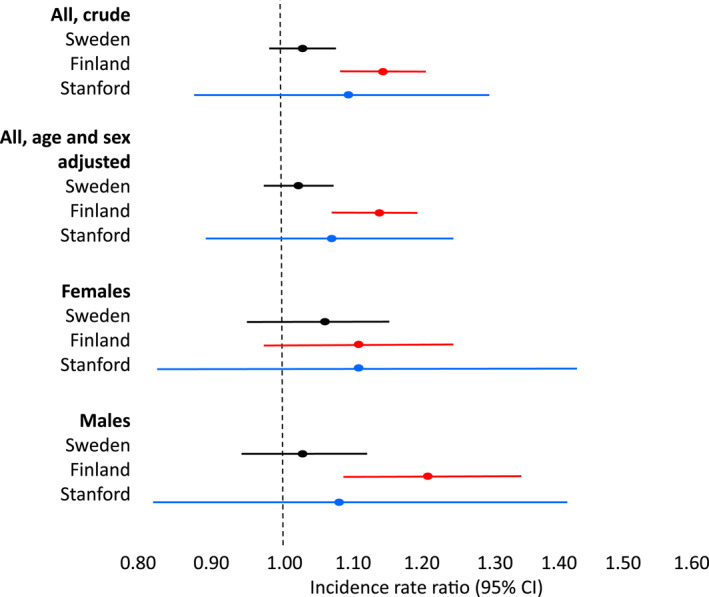
Incidence rate ratios (95% CI) of type 1 diabetes in children under the age of 15 years during the COVID‐19 lockdown compared with the reference period in Sweden, Finland, and Stanford, CA, the USA.

**FIGURE 2:**
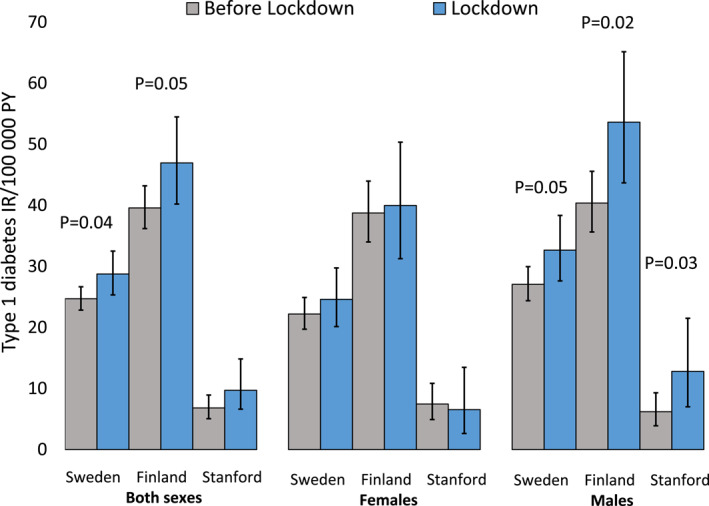
Type 1 diabetes incidence rates and 95% CIs in children under 5 years of age before and during COVID‐19 lockdown in Sweden, Finland, and Stanford, CA, the USA. Significant *p*‐values for IRRs are presented.

Type 1 diabetes incidence increased by 5% in Sweden (IRR = 1.05; 95% CI = 0.98–1.12; *p* = 0.18, *N* = 4458), 17% in Finland (IRR = 1.17; 95% CI = 1.07–1.27; *p* < 0.001, *N* = 2881), and 10% in Stanford (IRR = 1.10; 95% CI = 0.91–1.34; *p* = 0.34, *N* = 531) during the lockdown compared to the time before lockdown (Figure 1). In Finland, the difference was significant for boys (IRR = 1.20; 95% Cl = 1.08–1.34; *p* < 0.001, *N* = 1643) but not for girls (IRR = 1.12; 95% Cl = 0.99–1.27; *p* = 0.08, *N* = 1238) (Figure 2). In Sweden and Stanford, the incidence rate ratios were not significantly different when the two sexes were analysed separately (*p* values > 0.05, Table S1).

In the sensitivity analysis where we compared the lockdown period with only the most recent preceding 18‐month period before the lockdown, the incidence of type 1 diabetes was 39.3 per 100,000 person–years (95% CI 37.0–41.8) in Sweden, 51.2 per 100,000 person–years (95% CI 47.4–55.2) in Finland and 16.4 per 100,000 person–years (95% CI 13.7–19.6) in Stanford. The incidence during the lockdown was significantly higher compared with the most recent control period in Sweden (IRR = 1.10; 95% CI 1.01–1.19; *p* = 0.03) and Finland (IRR = 1.19; 95% CI 1.07–1.32; *p* = 0.009) but did not reach significance in Stanford (IRR = 1.15; 95% CI 0.90–1.47; *p* = 0.26).

### 3.2 Incidence Rates and Incidence Rate Ratios—Different Age Groups and Sex

**FIGURE 3:**
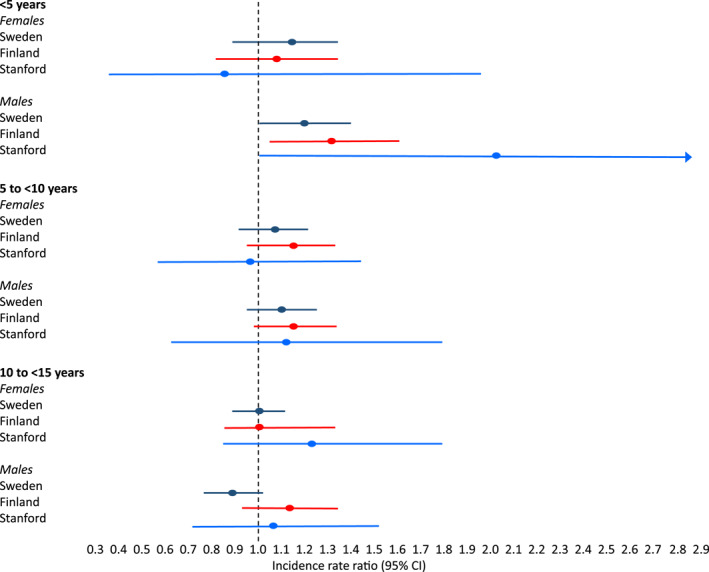
Incidence rate ratios (95% CI) of type 1 diabetes in children in three age groups during the COVID‐19 lockdown compared with the reference period in Sweden, Finland, and Stanford, CA, the USA. Data shown as rate (95% Cl).

The increase in type 1 diabetes incidence was significant in children < 5 years of age in Sweden (IRR = 1.16; 95% Cl = 1.01–1.34; *p* = 0.04, *N* = 885) and in Finland (IRR = 1.19; 95% Cl = 1.00–1.41; *p* = 0.05, *N* = 675) but not in Stanford CA, USA (IRR = 1.42; 95% CI = 0.86–2.37; *p* = 0.17, *N* = 71). The increase was significant in boys < 5 years of age in all three study locations: Sweden IRR = 1.21; 95% Cl = 1.00–1.46; *p* = 0.05, *N* = 521), Finland (IRR = 1.33; 95% Cl = 1.06–1.67; *p* = 0.02, *N* = 363) and Stanford CA, USA (IRR = 2.07; 95% Cl = 1.06–4.02, *p* = 0.03, *N* = 37) but not for girls < 5 years of age in any of the study locations (Figure 3). The incidence of type 1 diabetes increased in Finnish children 5–9 years of age (IRR = 1.18; 95% Cl = 1.04–1.34; *p* = 0.01, *N* = 1136) but not when the sexes were analysed separately (Table 1). Further sex‐specific analyses for different age groups did not reveal any additional differences between cohorts in any of the three study locations (Figures 1, 2, 3, Table 1, Table S1).

**TABLE 1:** Incidence rates of type 1 diabetes in children younger than 15 years old per 100,000 person‐years before and during the lockdown in Sweden, Finland, and Stanford, CA, the USA.

	Sweden	Sweden	Finland	Finland	Stanford	Stanford
	Before lockdown	Lockdown	Before lockdown	Lockdown	Before lockdown	Lockdown
All	41.3 (39.9–42.7) *N* = 3268	43.2 (40.8–45.7) *N* = 1190	52.3 (50.1–54.8) *N* = 2096	61.0 (56.8–65.4) *N* = 785	17.2 (15.5–19.0) *N* = 394	18.9 (11.9–26.4) *N* = 137
Girls	38.4 (36.5–40.4) *N* = 1477	40.9 (37.5–44.5) *N* = 547	46.5 (43.5–49.6) *N* = 911	52.0 (46.5–56.0) *N* = 327	17.5 (15.1–20.1) *N* = 196	19.4 (15.1–24.6) *N* = 69
Boys	44.0 (42.0–46.1) *N* = 1791	45.3 (41.9–49.0) *N* = 643	57.8 (54.6–61.2) *N* = 1185	69.6 (63.4–76.3) *N* = 458	16.9 (14.6–19.4) *N* = 198	18.4 (14.3–23.3) *N* = 68
Age < 5	24.7 (22.9–26.7) *N* = 660	28.7 (25.3–32.5) *N* = 255	39.6 (36.2–43.2) *N* = 502	47.0 (40.2–54.5) *N* = 173	6.9 (5.1–8.9) *N* = 50	9.7 (6.6–14.8) *N* = 21
Age < 5 girls	22.2 (19.7–24.9) *N* = 288	24.6 (20.14–29.75) *N* = 106	38.7 (34.0–44.0) *N* = 240	40.0 (31.3–50.3) *N* = 72	7.5 (4.9–10.8) *N* = 27	6.5 (2.6–13.5) *N* = 7
Age < 5 boys	27.1 (24.4–30.0) *N* = 372	32.7 (27.6–38.4) *N* = 149	40.4 (35.6–45.6) *N* = 262	53.6 (43.7–65.2) *N* = 101	6.2 (3.9–9.3) *N* = 23	12.8 (7.0–21.5) *N* = 14
Age 5 to < 10	43.1 (40.7–45.7) *N* = 1164	47.2 (42.9–51.8) *N* = 440	59.3 (55.3–63.5) *N* = 823	70.0 (62.4–78.2) *N* = 313	19.9 (16.9–23.2) *N* = 156	17.3 (12.5–23.2) *N* = 43
Age 5 to < 10 girls	43.9 (40.4–47.7) *N* = 576	47.5 (41.4–54.3) *N* = 215	52.8 (47.5–58.5) *N* = 358	62.2 (52.2–73.6) *N* = 136	20.5 (16.2–25.6) *N* = 78	19.7 (12.6–29.3) *N* = 24
Age 5 to < 10 boys	42.4 (39.0–45.9) *N* = 588	46.9 (40.9–53.4) *N* = 225	65.6 (59.7–71.8) *N* = 465	77.4 (66.4–89.6) *N* = 177	13.0 (10.6–15.8) *N* = 78	14.9 (9.0–23.3) *N* = 19
Age 10 to < 15	56.7 (53.8–59.7) *N* = 1444	52.9 (48.3–57.8) *N* = 495	57.0 (53.1–61.2) *N* = 771	63.5 (56.5–71.1) *N* = 299	24.3 (20.9–28.0) *N* = 188	28.1 (22.0–35.4) *N* = 73
Age 10 to < 15 girls	49.5 (45.7–53.6) *N* = 613	49.8 (43.5–56.7) *N* = 226	47.3 (42.2–52.9) *N* = 313	51.7 (42.8–61.9) *N* = 119	24.1 (19.4–29.6) *N* = 91	30.2 (21.3–41.4) *N* = 38
Age 10 to < 15 boys	63.5 (59.2–67.9) *N* = 831	55.8 (49.4–62.9) *N* = 269	66.3 (60.3–72.6) *N* = 458	74.8 (64.2–86.5) *N* = 180	24.4 (19.8–29.8) *N* = 97	26.2 (18.2–36.4) *N* = 35

*Note:* Data shown as rate (95% Cl).

### 3.3 Incidence Rates in Different Areas

The overall incidence of type 1 diabetes was highest in Finland (IR = 54.4 per 100,000 person years; 95% CI = 52.4–56.4, *N* = 2881), followed by Sweden (IR = 41.8 per 100,000 person years; 95% CI = 40.5–43.0, *N* = 4458) and lowest in Stanford, CA, USA (IR = 17.6 per 100,000 person years; 95% CI = 16.1–19.2, *N* = 531) (all area comparison *p* values < 0.001) in children under the age of 15 years during the two study periods combined (before lockdown and lockdown).

## 4 Discussion

Following our expectations, the rates of type 1 diabetes increased least in Sweden where only mild and voluntary social distancing regulations were implemented during the pandemic study period. However, type 1 diabetes incidences increased only marginally and non‐statistically also in Stanford, California, where very strict lockdown actions were implemented. Only in Finland, where lockdown actions were moderate, did type 1 diabetes incidence rates increase significantly. As observational research does not justify causal reasoning, explanations to these findings should be established using further intervention studies. However, in the following, we aim to put the current observations in context by reflecting them in relation to the existing scientific literature.

Some studies have shown no association between acute SARS‐CoV‐2 infections and subsequent type 1 diabetes in children [7, 17, 18], and islet autoimmunity or type 1 diabetes rates did not increase after SARS‐CoV‐2 infections in a follow‐up study of children carrying increased genetic susceptibility to type 1 diabetes [19]. We observed in an earlier study in Finland that less than 1% of children diagnosed with type 1 diabetes during the 18 first months of the COVID‐19 pandemic tested positive for SARS‐CoV‐2 antibodies combined with a simultaneous 17% increase in the incidence of type 1 diabetes [9]. Therefore, it is highly unlikely that COVID‐19 infection would have directly caused type 1 diabetes in children in Finland [9].

Similar to Finland, a significant increase in the incidence of type 1 diabetes has been observed in various countries during pandemic lockdowns [6]. COVID‐19 lockdowns resulted in radical changes in children's everyday lives, which, in turn, have been proposed to affect the incidences of type 1 diabetes in children [9]. For example, lockdowns led to changes in diet including decreased consumption of vegetables and fruits [20], changes in the amount and quality of daily physical activity [21, 22], an increase in body weight [23], mental health problems [24], increased duration but poor quality of sleep [25] and increased time spent using electronic devices such as tablets and TV [24].

Out of these factors, those related to nutrition [26], physical activity [27] and weight gain [28] have been associated with the risk of type 1 diabetes. Weight gain and obesity result in *β*‐cell stress by increasing the need of endogenous insulin to compensate for increasing plasma glucose concentrations. Physical activity increases the number of glucose transporters in the cell membranes leading to higher glucose uptake in skeletal muscles [29]. In addition, exercise could modulate the cytokines in an anti‐inflammatory direction and prevent *β*‐cell destruction [30]. Furthermore, more physical activity may result in lower BMI and reduce visceral fat mass (a source of fat‐derived cytokines) [31], which in turn could provide protection from type 1 diabetes. In addition, obesity may trigger chronic low‐grade inflammation and reduce the number of regulatory *B* and *T* cells. This has been proposed to lead to an increased activity of Th17 and Th1 cells, and thereby, to *β*‐cell destruction [32]. Consumption of some vegetables may provide protection from type 1 diabetes, for example, by influencing the gut microbiota and/or BMI [26].

Our findings do not systematically align with the hygiene hypothesis stating that childhood infections may protect children from type 1 diabetes. In addition to social distancing, lockdown measures that were particularly stringent in Stanford and Finland but much less so in Sweden included face mask usage and increased frequency of hand washing accompanied with usage of hand sanitizers [1, 2, 3, 4]. These actions have been shown to considerably reduce the contagion of respiratory tract infections [1, 2, 3, 4]. There was a sharp decrease in child respiratory syncytial virus (RSV), influenza and viral gastroenteritis during the winter 2020–2021 in many countries including Finland [3, 33]. In line with the hygiene hypothesis, the incidence of type 1 diabetes increased rapidly when the infection load decreased.

However, in Stanford, more stringent social distancing did not result in an increased incidence of type 1 diabetes, as could be expected based on the hygiene hypothesis. Therefore, other explanations such as differences in demographic, genetic and social factors affecting the risk of type 1 diabetes in different study locations must be considered. Unlike Finland and Sweden, the United States does not have a national level database for type 1 diabetes; thus, data can only be obtained from individual hospitals. Furthermore, the sample obtained from Stanford was smaller compared to the Swedish and Finnish data sets. In addition, the overall incidence of type 1 diabetes was, by far, lowest in Stanford out of the three locations included. Furthermore, referral patterns at Stanford may have varied over time, introducing uncertainty around incidence rates.

One limitation is that we were not able to assess the possible impact of the ethnic background on the association between the stringency of the lockdown actions and the rate of type 1 diabetes because of the lack of such data. Analyses of federal, state and local data have shown that Hispanic, Latino and African American people experienced higher rates of COVID‐19 cases compared with White subjects in the Stanford area and other parts of the USA during the pandemic [34, 35]. These ethnic groups may have had more social encounters during the pandemic, and thereby, more contacts with not only with SARS‐CoV‐2 but also other pathogens, and microbes compared to Caucasians. As the Hispanic minority makes approximately 25% of the total population in the Stanford area, this could have contributed to some of our findings; if the hygiene hypothesis holds true, people of colour may have had a lower risk of type 1 diabetes compared to Caucasians during COVID‐19 lockdowns. This may mask the association between the lockdown and risk of type 1 diabetes in our Stanford data set.

Daycare closures may explain why the increase in type 1 diabetes was most evident in children < 5 years of age in Finland and Sweden. Generally, children who attend daycare are two to three times more likely to acquire infections than children who do not attend such services [36]. In line with the hygiene hypothesis, several studies have revealed a significant protective association of daycare with the risk of type 1 diabetes in children [37, 38]. The incidence of type 1 diabetes increased significantly in this age group during the first 18 months of the pandemic also in Sweden, where daycare centres were operating as usual throughout the pandemic. Even so, a high proportion of Swedish parents kept their children at home during the first year of the pandemic [2, 39]. In addition, parents of young children were instructed to keep them at home even if they had very mild symptoms of any disease. Furthermore, parents were not allowed to enter the daycare facilities during the lockdown. All this may have resulted in substantially reduced exposure to infectious microbes, which in turn might have increased the risk of type 1 diabetes in this age group.

In the youngest age group, the increase in the rate of type 1 diabetes incidence was systematically found only in boys in all study locations. Girls typically have stronger immune responses to antigens than males, resulting in sex‐based differences in the risk of infectious diseases [40]. In both humans and animals, males are generally more susceptible than females to bacterial, viral, and fungal infections [40]. This may have resulted in a more pronounced reduction in the infection rates in boys during lockdowns. If the hygiene hypothesis holds true, this could have been reflected as a higher increase in the incidence of type 1 diabetes among boys.

Furthermore, the interplay between sex hormones and the immune system may contribute to the sex‐specific risk of developing type 1 diabetes. Prepubertal girls are shown to have higher IGF‐I, oestradiol, testosterone and leptin concentrations compared with boys [41]. Leptin levels are low in newly diagnosed patients with type 1 diabetes and increase after the institution of insulin therapy [42]. Oestrogens, in turn, have been proposed to protect pancreatic *β*‐cells by activating oestrogen receptor *α* (ERα) [43]. Furthermore, research on mice models and humans has shown that oestrogen has antidiabetic functions [44].

Our results do not align with the conception that contact with nature would protect from type 1 diabetes. Finnish people spent 89% and Swedish 64% more time outdoors during the lockdown compared to a reference time before the lockdown [45, 46]. In addition, the duration of outdoor playtime peaked in Finnish children during the lockdown in 2020 in a 3‐year repeated cross‐sectional study [22]. In Sweden, daycare centres were open, but activities were systematically moved to outdoor premises [39]. People in the Stanford area spent 5%–6% more time outdoors during the COVID‐19 pandemic compared to the time before the pandemic [47]. As the time spent outdoors increased, the incidence of type 1 diabetes increased or remained unchanged in our three study locations.

Our research is in line with national data showing that the incidence of type 1 diabetes is highest in Finland, second highest in Sweden and lowest in Stanford, CA, USA of the three study locations included [48, 49, 50]. The differences in incidence rates are in line with data from national statistics on type 1 diabetes incidence. In fact, Finland has by far the highest type 1 diabetes incidence in the world, whereas Sweden holds the second place in the global statistics. The reasons behind the varying incidence rates in different countries remain open. In addition to increased hygiene, several mechanisms including effects of diet, vitamin D deficiency, lack of breastfeeding, and enterovirus infections have been proposed [10]. Our current research indicates that the number of social interactions could also explain some of the observed differences. For example, Finnish children start daycare at a later age than their peers in many other countries [51].

One strength of our study is that it covered more than 90% of all children and adolescents who presented with type 1 diabetes during the study periods in Finland and Sweden. Another strength is that the research was conducted in three different areas, which allows for more generalisable inferences. However, Stanford data has some limitations, including a significantly smaller dataset that was obtained from individual hospitals instead of national diabetes registers like in Finland and Sweden. In addition, the Stanford data set likely did not cover all children living in the local counties since there are other diabetes centres in the region and health insurance often dictates at which hospital a child can receive care. Other limitations, including the inability to assess the possible impact of the ethnic background on our findings, were mentioned earlier in the discussion.

In conclusion, our results do not support the initial hypothesis that stricter lockdown actions would be associated with an increased risk of type 1 diabetes in the whole study population of children. However, lockdown actions during a pandemic may have untoward consequences such as an increased risk of type 1 diabetes in young children, boys in particular. The hygiene hypothesis may explain our findings, but other explanations cannot be excluded.

## Author Contributions

**Susanna Tall:** writing the original draft, methodology, investigation, formal analysis, data curation, conceptualization. **Priya Prahalad:** methodology, investigation, formal analysis, data curation, conceptualization. **Martin Adiels:** writing and editing, methodology. **Annika Rosengren:** writing and editing, methodology **Suvi M. Virtanen:** supervision, methodology, writing and editing, resources. **David M. Maahs:** writing and editing, validation, supervision, conceptualization. **Mikael Knip:** writing and editing, validation, supervision, methodology, conceptualization.

## Consent

All authors give full consent for publication.

## Conflicts of Interest

The authors declare no conflicts of interest.

## Peer Review

The peer review history for this article is available at https://www.webofscience.com/api/gateway/wos/peer-review/10.1002/dmrr.70084.

## Supporting information


**Table S1**: Incidence rate ratios (95% CI) of type 1 diabetes in children under the age of 15 years during the COVID‐19 lockdown compared with the reference period in Sweden, Finland, and Stanford, CA, the USA.

## Data Availability

Data may be available for a reasonable request to the corresponding author.
