# Supplementary material for: Increased Risk of Type 1 Diabetes in Boys Under the Age of 5 Years During COVID‐19 Lockdowns in Finland, Sweden and Stanford, CA, USA—An Observational Multicenter Study
Source: Diabetes Metab Res Rev. 2025 Sep 1;41(6):e70084. doi: 10.1002/dmrr.70084 (PMC12400900; doi:10.1002/dmrr.70084)
Supplement: Supplementary file 1 — Table S1: Incidence rate ratios (95% CI) of type 1 diabetes in children under the age of 15 years during the COVID‐19 lockdown compared with the reference period in Sweden, Finland, and Stanford, CA, the USA. [file DMRR-41-e70084-s001.docx]

**Supplementary Table S1.** Incidence rate ratios (95% CI) of type 1 diabetes in children under the age of 15 years during the COVID-19 lockdown compared with the reference period in Sweden, Finland, and Stanford, CA, the USA.

| **Area** | **Sex** | **Age group** | **Control**  **T1D cases (N)** | **Lockdown**  **T1D cases (N)** | **IRR** | **95% CI Lower** | **95% CI Upper** | **p-value** |
| --- | --- | --- | --- | --- | --- | --- | --- | --- |
| **Finland** | **both** | All | 2096 | 785 | 1.17 | 1.07 | 1.27 | **<.001** |
|  |  | 0-4 | 502 | 173 | 1.19 | 1.00 | 1.41 | **0.053** |
|  |  | 5-9 | 823 | 313 | 1.18 | 1.04 | 1.34 | **0.013** |
|  |  | 10-14 | 771 | 299 | 1.11 | 0.97 | 1.27 | 0.114 |
|  | **female** | All | 911 | 327 | 1.12 | 0.99 | 1.27 | 0.083 |
|  |  | 0-4 | 240 | 72 | 1.03 | 0.79 | 1.34 | 0.816 |
|  |  | 5-9 | 358 | 136 | 1.18 | 0.97 | 1.44 | 0.103 |
|  |  | 10-14 | 313 | 119 | 1.09 | 0.88 | 1.35 | 0.411 |
|  | **male** | All | 1185 | 458 | 1.20 | 1.08 | 1.34 | **0.001** |
|  |  | 0-4 | 262 | 101 | 1.33 | 1.06 | 1.67 | **0.016** |
|  |  | 5-9 | 465 | 177 | 1.18 | 0.99 | 1.40 | 0.061 |
|  |  | 10-14 | 458 | 180 | 1.13 | 0.95 | 1.34 | 0.171 |
| **Sweden** | **both** | All | 3268 | 1190 | 1.05 | 0.98 | 1.12 | 0.179 |
|  |  | 0-4 | 660 | 225 | 1.16 | 1.01 | 1.34 | **0.040** |
|  |  | 5-9 | 1164 | 440 | 1.09 | 0.98 | 1.22 | 0.107 |
|  |  | 10-14 | 1444 | 495 | 0.93 | 0.84 | 1.03 | 0.184 |
|  | **female** | All | 1477 | 547 | 1.07 | 0.97 | 1.18 | 0.206 |
|  |  | 0-4 | 288 | 106 | 1.11 | 0.89 | 1.38 | 0.366 |
|  |  | 5-9 | 576 | 215 | 1.08 | 0.93 | 1.27 | 0.323 |
|  |  | 10-14 | 613 | 226 | 1.01 | 0.86 | 1.17 | 0.947 |
|  | **male** | All | 1791 | 643 | 1.03 | 0.94 | 1.13 | 0.506 |
|  |  | 0-4 | 372 | 149 | 1.21 | 1.00 | 1.46 | **0.052** |
|  |  | 5-9 | 588 | 225 | 1.11 | 0.95 | 1.29 | 0.197 |
|  |  | 10-14 | 831 | 269 | 0.88 | 0.77 | 1.01 | 0.068 |
| **Stanford** | **both** | All | 394 | 137 | 1.10 | 0.91 | 1.34 | 0.339 |
|  |  | 0-4 | 50 | 21 | 1.42 | 0.86 | 2.37 | 0.174 |
|  |  | 5-9 | 156 | 43 | 0.87 | 0.62 | 1.22 | 0.415 |
|  |  | 10-14 | 188 | 73 | 1.16 | 0.88 | 1.52 | 0.289 |
|  | **female** | All | 196 | 69 | 1.11 | 0.84 | 1.46 | 0.450 |
|  |  | 0-4 | 27 | 7 | 0.88 | 0.38 | 2.02 | 0.758 |
|  |  | 5-9 | 78 | 24 | 0.96 | 0.61 | 1.52 | 0.871 |
|  |  | 10-14 | 91 | 38 | 1.25 | 0.86 | 1.82 | 0.249 |
|  | **male** | All | 198 | 68 | 1.09 | 0.83 | 1.43 | 0.551 |
|  |  | 0-4 | 23 | 14 | 2.07 | 1.06 | 4.02 | **0.032** |
|  |  | 5-9 | 78 | 19 | 0.77 | 0.47 | 1.28 | 0.315 |
|  |  | 10-14 | 97 | 35 | 1.07 | 0.73 | 1.58 | 0.725 |
